# Vaginal candidiasis attributed to hair, skin, and nails supplement: a case report

**DOI:** 10.1186/s13256-025-05217-y

**Published:** 2025-04-21

**Authors:** Faith Whitlock, Akram Al-Makki, Brian Shepler

**Affiliations:** 1 Purdue University College of Pharmacy, West Lafayette, USA; 2 IU Health Arnett Nephrology, Lafayette, USA

**Keywords:** Vaginal candidiasis, Supplements, Adverse drug event

## Abstract

**Background:**

This case report presents the first documented instance of vaginal candidiasis linked to the intake of a hair, skin, and nails supplement.

**Case presentation:**

A 64-year-old Caucasian female patient, with a history of chronic kidney disease and multiple comorbidities, developed symptoms of vaginal yeast infection after beginning the supplement. The adverse event was evaluated using the Naranjo Adverse Drug Reaction Probability Scale, scoring a 5, indicating a probable association. Despite extensive literature searches, no similar cases were found, suggesting this might be a unique reaction. This report highlights potential safety concerns with dietary supplements, particularly those not regulated by the Food and Drug Administration, underscoring the need for further investigation into their adverse effects.

**Conclusions:**

The case emphasizes the importance of considering dietary supplements as potential contributors to adverse health events, especially in patients with complex medical histories.

## Introduction

Dietary supplements are utilized to help provide essential nutrients that the body needs to function properly [1]. Among the various types of supplements, hair, skin, and nails supplements, which are marketed by various brands, often referred to as “beauty supplements” are growing rapidly in popularity [2]. The National Health and Nutrition Examination survey has reported a nearly twofold use in these products [3]. Direct-to-consumer advertising and social media may play a larger role in this increase than ever before [4]. These products typically contain a blend of vitamins, minerals, and nutrients aimed at enhancing the health and appearance of hair, skin, and nails. Hair, skin, and nails supplements are not regulated as drugs by the Food and Drug Administration (FDA) and as such they are not tested for safety and efficacy. They are instead recognized as foods by the Dietary Supplement Health and Education Act [5, 6]. While certain adverse events have been reported and include acute reactions such as sedation and kidney stones as well as chronic toxicities such as gastrointestinal upset and increased incidence of dyslipidemia and hyperglycemia, no reports of vaginal candidiasis have been published [4]. The following case report describes a patient taking a hair, skin, and nails supplement who developed vaginal candidiasis. Vaginal candidiasis is a fungal infection characterized by symptoms such as itching and irritation in the vagina and vulva, a burning sensation, redness and swelling of the vulva, vaginal pain, and a thick, white, odor-free vaginal discharge [7]. There are multiple ingredients in a hair, skin, and nails supplement including vitamins A, B vitamins, C, D, E; folate; zinc; rutin; pantothenic acid; para-aminobenzoic acid; horsetail extract; inositol; and collagen [8]. Each of these ingredients was included in the medical literature database searches described below to investigate the causative agents and degree of prevalence in the population. This adverse event was then assessed using the Naranjo Adverse Drug Reaction Probability Scale to determine the likelihood of causality.

## Case presentation

A 64-year-old Caucasian woman was seen in the nephrology clinic for the management of her chronic kidney disease on 7 June 2024. The patient’s past medical history was significant for stage 2 chronic kidney disease, hypertension, hyperlipidemia, arthritis, anemia of chronic kidney disease, fibromyalgia, peripheral vascular disease, gastroesophageal reflux disease, and urinary tract infections. Her estimated glomerular filtration rate (GFR) was > 60 ml/minute, blood pressure was 123/83 mmHg, and heart rate was 51 beats per minute. Her medications included albuterol 90 mcg hydrofluoroalkane (HFA) inhaler as needed, aspirin 81 mg daily, cholecalciferol 2000 IU daily, diclofenac 1% topical gel four times a day, docusate senna 2 capsule at bedtime, ezetimibe 10 mg daily, famotidine 40 mg at bedtime, ferrous sulfate 325 mg (65 mg elemental iron) daily, lisinopril 10 mg daily, metoprolol tartrate 100 mg twice daily, simvastatin 20 mg daily, and trazodone 200 mg at bedtime. The patient reported taking cranberry extract as well. Past medical history was positive for tobacco and negative for alcohol consumption. The patient started taking vitamin B12 and extra strength hair, skin, and nails supplement manufactured by Spring Valley in February after seeing the benefits of stronger nails and better skin from her cousin. The exact ingredients of Spring Valley Hair Skin and Nails supplement is listed in Table 1. After 3–4 weeks of taking the supplements daily, the patient started to experience dark, yellow, foul-smelling discharge from her vagina, and burning upon urination. The patient discontinued the supplement in mid-April and upon discontinuation, her symptoms improved.

**Table 1:** Hair, skin, and nails supplement ingredients per daily dose (three caplets)

Vitamin A	1500 mcg
Vitamin C	60 mg
Vitamin D	25 mcg
Vitamin E	6.75 mg
Thiamine	5 mg
Riboflavin	2 mg
Niacin	25 mg
Vitamin B6	5 mg
Folate	333 mcg
Vitamin B12	8 mcg
Biotin	3000 mcg
Pantothenic acid	15 mg
Choline	10 mg
Calcium	200 mg
Phosphorus	87 mg
Magnesium	50 mg
Zinc	7.5 mg
Manganese	2 mg
Para aminobenzoic acid	10 mg
Inositol	30 mg
Rutin complex	38 mg
Horsetail	3 mg
Collagen	50 mg

## Methods

The Naranjo Adverse Drug Reaction Probability Scale was used to assess the probability that the hair, skin, and nails supplement was associated with the patient’s vaginal candidiasis. The Naranjo Adverse Drug Reaction Probability Scale (Tables 2) ranges from −4 to +13. If a score is 9 or higher, it is considered “definite,” if the score is 5–8 it is “probable,” if the score is 1–4 it is “possible,” and if the score is 0 or less it is “doubtful” [9]. The patient consented to the use of her medical information for this work and was interviewed. Responses from the interview and from the patient’s medical history were recorded on the Naranjo Adverse Drug Reaction Probability Scale instrument. A literature search was conducted using the key terms “hair, skin, and nail supplement”, “yeast infection”, “urinary tract infection”, “vitamin B12 supplement”, “vaginal candidiasis”, and “beauty supplements”. Additionally, each ingredient was searched with vaginal candidiasis. These searches were completed using PubMed, Google Scholar, and Embase. The limits were set to include any papers published in human subjects in the English language. No limits were set to select for publication types or study designs.

**Table 2:**
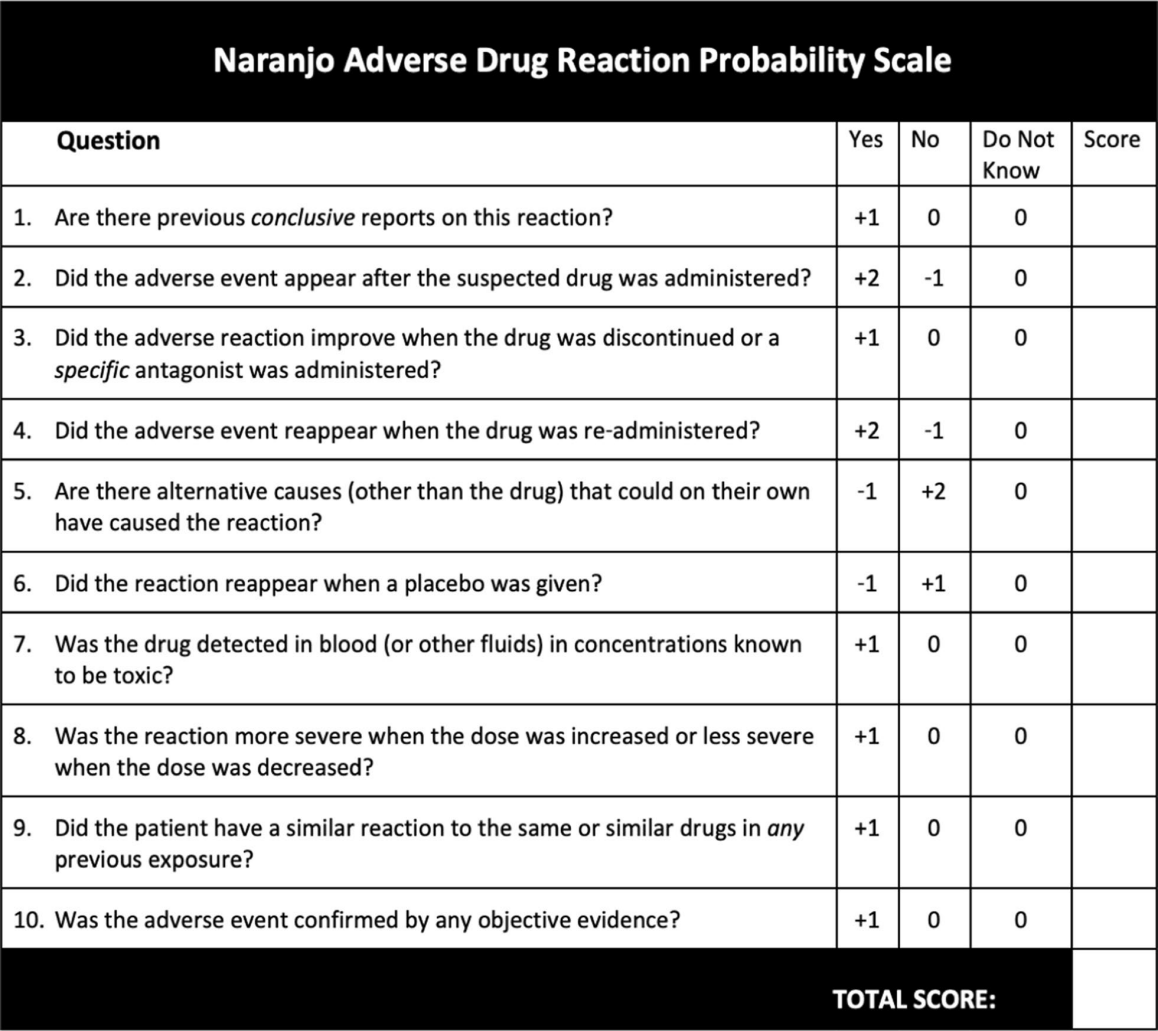
Naranjo adverse drug reaction probability scale [5]

Score interpretation

≥ 9 Definite: reaction (1) followed a reasonable temporal sequence after a drug or in which a toxic drug level had been established in body fluids or tissues, (2) followed a recognized response to the suspected drug, and (3) was confirmed by improvement on withdrawing the drug and reappeared on reexposure

5–8 Probable: reaction (1) followed a reasonable temporal sequence after a drug, (2) followed a recognized response to the suspected drug, (3) was confirmed by withdrawal but not exposure to the drug, and (4) could not be reasonably explained by the known characteristics of the patient’s clinical state

1–4 Possible: reaction (1) followed a temporal sequence after a drug, (2) possibly followed a recognized pattern to the suspected drug, and (3) could be explained by the characteristics of the patient’s disease

≤ 0 Doubtful: reaction was likely related to factors other than a drug

## Results

The Naranjo Adverse Drug Reaction Probability Scale reported a score of 5 for this adverse drug event, indicating probable cause (Table 3). The literature evaluation using the broad search methods described above returned no citations. No previous case reports on hair, skin, and nails supplements or their component ingredients as listed in the methods section observed vaginal candidiasis reactions.

**Table 3:** Patient score using Naranjo adverse drug reaction probability scale

Question	Score
1. Are there previous conclusive reports on this reaction?	No (+0)
2. Did the adverse event appear after the suspected drug was administered?	Yes (+2)
3. Did the adverse reaction improve when the drug was discontinued or a specific antagonist was administered?	Yes (+1)
4. Did the adverse event reappear when the drug was re-administered?	Do not know (+0)
5. Are there alternative causes (other than the drug) that could on their own have caused the reaction?	No (+2)
6. Did the reaction reappear when a placebo was given?	Do not know (+0)
7. Was the drug detected in blood (or other fluids) in concentrations known to be toxic?	No (+0)
8. Was the reaction more severe when the dose was increased or less severe when the dose was decreased?	Do not know (+0)
9. Did the patient have a similar reaction to the same or similar drugs in any previous exposure?	No (+0)
10. Was the adverse event confirmed by any objective evidence?	No (+0)
Total score	5: probable

## Discussion

The objective of this case report was to highlight the possible association between use of a common hair, skin, and nails supplement and the presence of vaginal candidiasis. To the authors’ knowledge, this is the first reported case of a hair, skin, and nails supplement associated with vaginal candidiasis. The importance of this case is that it highlights the possibility of links between common, readily available products that patients use every day without the realization of unintended side effects. In this case, the patient would not normally consider that hair, skin, and nails treatment could be affecting her genitourinary system. No formal studies or randomized controlled trials have evaluated the likelihood of developing vaginal candidiasis from the routine use of these products. Furthermore, manufacturers do not have to prove that hair, skin, and nails supplements are safe or effective as they are not regulated by the FDA [6]. This severely limits the knowledge of adverse effects and relegates the reporting of such instances to case reports and anecdotal accounts. All other medication related causes for vaginal candidiasis in this case were investigated. This patient was on several prescribed medications, none of which were reported to cause vaginal candidiasis. Other known causes such as uncontrolled diabetes and antibiotic use were also ruled out. Although age, hormonal changes, stress, and immunity issues can be contributing factors to vaginal candidiasis, the physician did not believe these were contributory in this case. The patient’s reaction appeared after she started taking the hair, skin, and nails supplement and discontinued when she stopped. How vaginal candidiasis could be attributed to a hair, skin, and nails supplement is difficult to ascertain. Although the active ingredients have not been reported to cause vaginal candidiasis, there could be other inactive ingredients or preservatives that may alter the vaginal pH with extended use. Limitations to this work include the fact that this is only one patient, and more reports or randomized studies would be needed for definitive conclusions. The patient also did not receive a comprehensive review of her dietary habits and thus no information is available regarding foods that might affect vaginal pH. It could be that many consumers are unaware that vaginal candidiasis could be linked to something as seemingly benign as a hair, skin, and nails supplement and hence many cases may go unreported.

## Conclusion

The patient case presented here reports an association between hair, skin, and nails and vitamin b12 supplementation and vaginal candidiasis. Hair, skin, and nails supplements, while increasing in popularity, are not regulated by the FDA as drugs and no controlled studies exist to evaluate their safe and effective use. This case report highlights one concern that may link this product to the development of vaginal candidiasis and is worth further investigation.

## Data Availability

Not applicable.

## References

[1] Bailey R, Gahche J, Lentino C, *et al*. Dietary supplement use in the United States, 2003–2006. J Nutr. 2011;141(2):261–6. 21178089 10.3945/jn.110.133025 PMC3021445

[2] Trepanowski N, Moore KJ, Kim DY, Hartman RI. Trends in hair, skin, and nails supplement use: data from the National Health and Nutrition Examination Survey (NHANES) 2011–2020. J Am Acad Dermatol. 2023. 10.1016/j.jaad.2023.02.026. (**Published online February 27, 2023**). 37348562 10.1016/j.jaad.2023.02.026

[3] Perez-Sanchez AC, Tantry EK, Burns EK, Perez VM, Prabhu S, Katta R. Skin, hair, and nail supplements: marketing and labeling concerns. Cureus. 2020;12(12): e12062. 10.7759/cureus.12062. (**Published 2020 Dec 13**). 33447491 10.7759/cureus.12062 PMC7802115

[4] Burns EK, Perez-Sanchez A, Katta R. Risks of skin, hair, and nail supplements. Dermatol Pract Concept. 2020;10(4): e2020089. 10.5826/dpc.1004a89. (**Published 2020 Oct 26**). 33150030 10.5826/dpc.1004a89 PMC7588165

[5] Katta R, Huang S. Skin, hair, and nail supplements: an evidenced-based approach. Skin Therapy Lett. 2019;24(5):7–13. 31584785

[6] Dietary Supplement Health and Education Act of 1994 Public Law 103-417 103rd Congress. NIH Office of Dietary Supplements. US Department of Health and Human Services. 1994. https://ods.od.nih.gov/About/DSHEA_Wording.aspx. Accessed Sept 2024.

[7] Mayo Clinic. “Yeast Infection (Vaginal) – Symptoms and Causes.” Mayo Clinic, 2019, www.mayoclinic.org/diseases-conditions/yeast-infection/symptoms-causes/syc-20378999.

[8] Hair, Skin, and Nails Dietary Supplement. Walmart. https://www.walmart.com/ip/Spring-Valley-Hair-Skin-Nails-Dietary-Supplement-Softgels-5-000-Mcg-Biotin-120-Ct/49929247. Accessed 10 Apr 2024.

[9] Naranjo CA, Busto U, Sellers EM, *et al*. A method for estimating the probability of adverse drug reactions. Clin Pharmacol Ther. 1981;30(2):239–45. 10.1038/clpt.1981.154. 7249508 10.1038/clpt.1981.154

